# Cosmetics and personal hygiene primary plastic packaging dataset: Characterization of post-consumer waste returned via take-back system in Denmark

**DOI:** 10.1016/j.dib.2025.112300

**Published:** 2025-11-23

**Authors:** Gloria Moscatelli, Heather Margaret Logan, Lærke Djurhuus Ipsen, Jakob Seidel Bisgaard, Jonathan Holger Harlev Astrup, Ricardo Gabbay de Souza, Marie Kampmann Eriksen, Anders Damgaard

**Affiliations:** a Department of Environmental and Resource Engineering, Technical University of Denmark, 2800 Kongens Lyngby, Denmark; b Centre for Absolute Sustainability, Technical University of Denmark, 2800 Kgs, Lyngby, Denmark

**Keywords:** Circular economy, Waste characterization, Industrial ecology, Polymers, Recycling, Waste management

## Abstract

Cosmetics and personal hygiene packaging play a critical role in the transition to a circular economy. These products are widely used and are increasingly targeted by regulatory frameworks such as the European Union Packaging and Packaging Waste Regulation, which emphasizes design-for-recycling, take-back systems, and producer responsibility.

Due to their diverse packaging designs and product contents, cosmetics and personal hygiene packaging present challenges for recycling and end-of-life management. These products must appeal to consumers, while also enabling safe delivery of the cosmetic product during the products lifetime and intended use case. This work investigates how aesthetic elements and delivery mechanisms influence the recyclability of packaging, through a waste composition analysis of collected packaging design and composition, as well as the presence of residual cosmetic content. This data is crucial to address growing policy attention to packaging design for recycling, and providing detailed data on packaging types, formats, and residual contents in waste streams today. This dataset supports accurate forecasts for collection yields, recycling capacities and recyclability assessments, as well as ensuring efficient planning for sorting infrastructure, design improvements, and circular economy policy development for plastic cosmetics and personal hygiene packaging.

The dataset presented in this work, was generated from approximately 270 kg of post-consumer cosmetics and personal hygiene primary plastic packaging collected via a company-managed retail take-back system in Denmark. The packaging waste, collected from 72 retail stores, was analyzed between March and May 2025. The dataset encompasses 6.503 individual packaging samples systematically categorized according to a four-tiered characterization approach by product brand ownership, packaging format, product content, and container colour. Each packaging sample was also weighed in its original returned (wet) state and again after cleaning (dry condition) to quantify residual product contents.

The dataset is structured across three Excel sheets: (1) a coding scheme for packaging design, (2) a four-tiered characterization approach used to categorize the packaging samples, and (3) a main dataset containing raw measurements and statistical summaries, including means and standard deviations.

Researchers, policymakers, industry stakeholders, and sustainability experts can use this dataset to inform precise forecasting, mapping, and analysis of recycling potentials and design practices. Moreover, the structured methodological approach serves as a replicable framework for generating comparable datasets across various geographical contexts, thus supporting broader efforts in transitioning cosmetic and personal hygiene packaging toward circularity.

Specifications Table

**Table:** 

Subject	Earth & Environmental Sciences
Specific subject area	Waste characterization of post-consumer cosmetics and personal hygiene packaging returned via company managed take-back systems.
Type of data	Filtered Excel
Data collection	Post-consumer cosmetics and personal hygiene packaging waste collected via a company-managed take-back system in Denmark was collected from 72 stores located across the country. After manual sorting, packaging, whose primary container was made of plastic, was grouped by packaging design and categorized based on label information, such as brand and product content, as well as physical characteristics, such as container colour. Samples were then weighed as collected, cleaned to remove any residual product and after drying, weighed to determine the residual content. For cases with more than three samples for a unique packaging type, three samples were used as representative of the dry weight, and the mean was used to calculate residual content in other samples.
Data source location	The samples were collected in Denmark, and all characterization and lab analysis were carried out in the Department of Resource and Environmental Engineering’s Waste Laboratory at the Technical University of Denmark. The dataset is stored in the open-source data repository DTU Data, which is managed by the Technical University of Denmark.
Data accessibility	Repository name: DTU Data Data identification number: 10.11583/DTU.29856608
Related research article	None

## 1 Value of the Data

• Provides a detailed waste characterization of post-consumer cosmetics and personal hygiene plastic packaging collected via a company-managed take-back system in Denmark, offering insights into product characteristics such as container and closure designs, average weights and trends in residual content.
• Supports the analysis of the waste treatment needs for common cosmetics and personal hygiene packaging and its suitability for circularity and eco-design initiatives based on their residual content and physical characteristics.
• Supports the assessment of opportunities for design improvements and the suitability of cosmetics and personal hygiene packaging to for example: reduce residual content, improve waste sorting and recycling efficiency.
• Supplies a representative dataset for developing life cycle inventories of cosmetics and personal hygiene packaging for use in environmental modelling such as Life Cycle Assessment and Material Flow Analysis, enabling the evaluation of the associated resource flows and impacts.

## 2 Background

Upcoming Extended Producer Responsibility (EPR) regulations under the EU Packaging and Packaging Waste Regulation establish mandatory producer-funded end-of-life management and set a 55 % recycling target for plastic packaging by 2030, while promoting take-back schemes and design-for-recycling guidelines to advance high-quality material recovery [1,2]. While plastic packaging makes up around 39 % of plastic conversion occurring in the EU [3], it accounts for >59 % of plastic waste generation in the EU [4]. Due to contamination from residual content and a lack of homogeneity in collected post-consumer waste streams, only 12.6 % of all plastic products in the European market include post-consumer recycled content [2,3]. Unlike food packaging, cosmetic and personal-hygiene packaging often contains high-value contents and exhibits longer use life with the consumer, making it an ideal target for producer take-back schemes [5]. However, detailed publicly available data on the packaging design characteristics of cosmetics and personal-hygiene products are lacking, as well as residual content information [5]. This dataset samples ∼270 kg of post-consumer cosmetics and personal-hygiene packaging returned via a retail take-back system in Denmark and provides an objective baseline to support stakeholders in modelling environmental impacts, developing circular strategies, and addressing policy targets. The take-back system allows customers to return primary plastic packaging from cosmetic and personal-care products, including items from both in-house product lines and external brands. Used containers can be handed in at the store counter or placed in dedicated collection bins. As participation is entirely voluntary and not supported by any deposit or financial incentive, the system depends largely on consumer awareness and individual motivation to support recycling efforts.

## 3 Data Description

The dataset contains a detailed characterization of post-consumer cosmetics and personal hygiene packaging waste collected through a company-managed take-back system in Denmark. It captures packaging design, label information, and physical characteristics of the packaging, with a focus on parameters relevant to recyclability.

These data are intended to support researchers, industry stakeholders, and policymakers working in the fields of design, waste management, and circular economy transitions of cosmetic and personal hygiene packaging product systems. The information provided can be readily used to inform the development of Life Cycle Inventories and Material Flow Analysis, as well as the identification of circularity hotspots such as high-residue packaging or formats that are difficult to sort with current recycling systems. While the data were collected in Denmark and therefore best represent this geography, the packaging categorization approach and the physical measurements recorded can provide valuable insights into design and end-of-life aspects of cosmetics and personal hygiene packaging. The dataset was quality-checked for entry errors, formatting inconsistencies, and outliers, and standardized terminology was applied across all classification fields.

The final dataset is available via the DTU Data repository, DOI http://doi.org/10.11583/DTU.29856608 [6].

The single dataset file “Full Dataset” contains all data in Excel format. This file has been further split into five CSV files for easier use in code, where the first three are explained in detail below: S1 - Packaging design; S2 - Characterization tiers; S3 - Waste characterization. The main variables are furthermore detailed in the *Read Me* file. Photos of the packaging design are provided in *Supplementary Material S1: Packaging Design*. In addition, a table containing the geographical details of the stores from which the cosmetics and personal hygiene plastic packaging waste was collected is included in the file *Store Locations*.

### 3.1 S1 - Packaging design

The Excel sheet “S1 - Packaging design” presents the standardized codes and corresponding code description for primary container types and closure mechanisms. Key variables are reported in Table 1. The Excel sheet serves as a reference for interpreting codes applied throughout the dataset and ensures consistency in packaging format identification.

**Table 1:** Column headings and description of the variables in S1 - Packaging design Dataset. The table is also presented in the Read Me document.

Variable	Description
**Primary container type - CODE**	Code assigned to each container type
**Primary container type**	Text label describing the primary container type
**Closure mechanism type - CODE**	Code assigned to each closure type
**Closure mechanism type**	Text label describing the closure type

### 3.2 S2 Characterization tiers

The Excel file S2 - Characterization tiers, defines a four-tiered classification system used to categorize each sample in the final dataset. Key variables are reported in Table 2. The classification system allows for a structured analysis of brand ownership (Tier I), packaging design (Tier II), product content and associated emptying efficiency (Tier III), and container colour (Tier IV). It was derived from a retailer-specific product catalogue and expert judgment in the absence of a standardized industry scheme for packaging formats. Each sample was assigned to a category within each tier, enabling structured grouping and subsequent analysis.

**Table 2:** Column headings and description of the variables in S2 – Characterization tiers. The table is also presented in the Read Me document.

Variable	Description
**Tier**	Classification level (I to IV)
**Category**	Main grouping type for each tier (e.g. brand ownership, packaging design)
**Category specification**	Sub-definition of the main grouping type (e.g. Container type), if applicable
**Subcategory**	Individual entries (e.g. Bottle, Black)

### 3.3 S3 - Waste characterization

The primary dataset is in Sheet S3 – Waste characterization, where each row corresponds to a single sample. Key variables are reported in Table 3. The dataset includes both raw measurements and calculated fields. The final dataset includes 6503 sampled packaging units, covering 823 unique packaging types across 227 brands.

**Table 3:** Column headings and description of the variables in S3 – Waste characterization. The table is also presented in the Read Me document.

Variable	Description
**Group ID**	Sample-level grouping identifiers
**Subgroup ID**	Sub-definition of the group ID
**Brand name**	Brand of the sample as noted on the packaging
**Sample content**	Declared content of the sample - see full list in tier III in the ``S1 - Characterization tiers'' Excel sheet
**Primary container type - CODE**	Structural packaging format - see full list in tier II in the ``S1 - Characterization tiers'' Excel sheet
**Closure mechanism type - CODE**	Sealing or dispensing mechanism - see full list in tier II in the ``S1 - Characterization tiers'' Excel sheet
**Missing component**	Missing parts of the packaging samples at the time of measurement. MB = Missing base of the container; MC = missing closure; MO = missing overcap; MT = Missing top (both closure and overcap)
**Declared volume**	Volume of product content as stated on the packaging
**Unit**	Specific unit, milliliters [ml], grams [g] or pieces [pcs], used to define the declare volume
**Container colour**	Container body colour - see full list in tier IV in the ``S1 - Characterization tiers'' Excel sheet
**Total wet packaging weight**	Total weight of each sample, including leftover product, before cleaning; measured in grams.
**Dry packaging weight - Mean**	Mean total weight of dried packaging (including container, closure, labels, etc.), calculated from triplicate measurements when applicable; measured in grams.
**Dry packaging weight - Std**	Standard deviation of the dry weight measurements; measured in grams.
**Residual content - Mean**	Mean of residual content for a group of identical samples - based on weight difference between wet and dry sample, indicating leftover product; measured in grams.
**Residual content - Std**	Standard deviation of the residual content; measured in grams.
**Normalized residual content**	Residual content normalized by declared product volume; measured in grams per millilitre.
**Container weight**	Weight of the container calculated as the difference between the dry packaging weight and the closure and other parts; measured in grams.
**Closure weight - Mean**	Mean weight of the closure (e.g. cap, pump), calculated from triplicate measurements when applicable; measured in grams.
**Closure weight - Std**	Standard deviation of the closure weights; measured in grams.
**Pump weight - Mean**	Mean weight of pump closure, calculated from triplicate measurements when applicable; measured in grams.
**Pump weight - Std**	Standard deviation of pump weights; measured in grams.
**Label weight - Mean**	Mean weight of product labels, calculated from triplicate measurements when applicable; measured in grams.
**Label weight - Std**	Standard deviation of label weights; measured in grams.
**Additional component type**	Description of any additional component (e.g. plastic seal).
**Additional component weight - Mean**	Mean weight of the additional components, calculated from triplicate measurements when applicable; measured in grams.
**Additional component weight - Std**	Standard deviation of weights for the additional component; measured in grams.
**Group ID**	Sample-level grouping identifiers
**Subgroup ID**	Sub-definition of the group ID
**Brand name**	Brand of the sample as noted on the packaging
**Sample content**	Declared content of the sample - see full list in tier III in the ``S1 - Characterization tiers'' Excel sheet
**Primary container type - CODE**	Structural packaging format - see full list in tier II in the ``S1 - Characterization tiers'' Excel sheet
**Closure mechanism type - CODE**	Sealing or dispensing mechanism - see full list in tier II in the ``S1 - Characterization tiers'' Excel sheet

## 4 Experimental Design, Materials and Methods

The sampling of post-consumer cosmetic and personal-hygiene packaging collected via a retail take-back scheme in Denmark was conducted in partnership with the retailer. For this campaign, the sampling included products returned to 72 different outlets in the peninsula of Jutland and the Danish archipelago (excluding Bornholm, Greenland, and the Faroe Islands) from February to March of 2025, resulting in >270 kg of material for testing. At its core, the takeback system is intended to ensure producer responsibility for their own in-house goods, but to ease the convenience for the consumer, they accept all brands and products they sell.

**Fig. 1:**
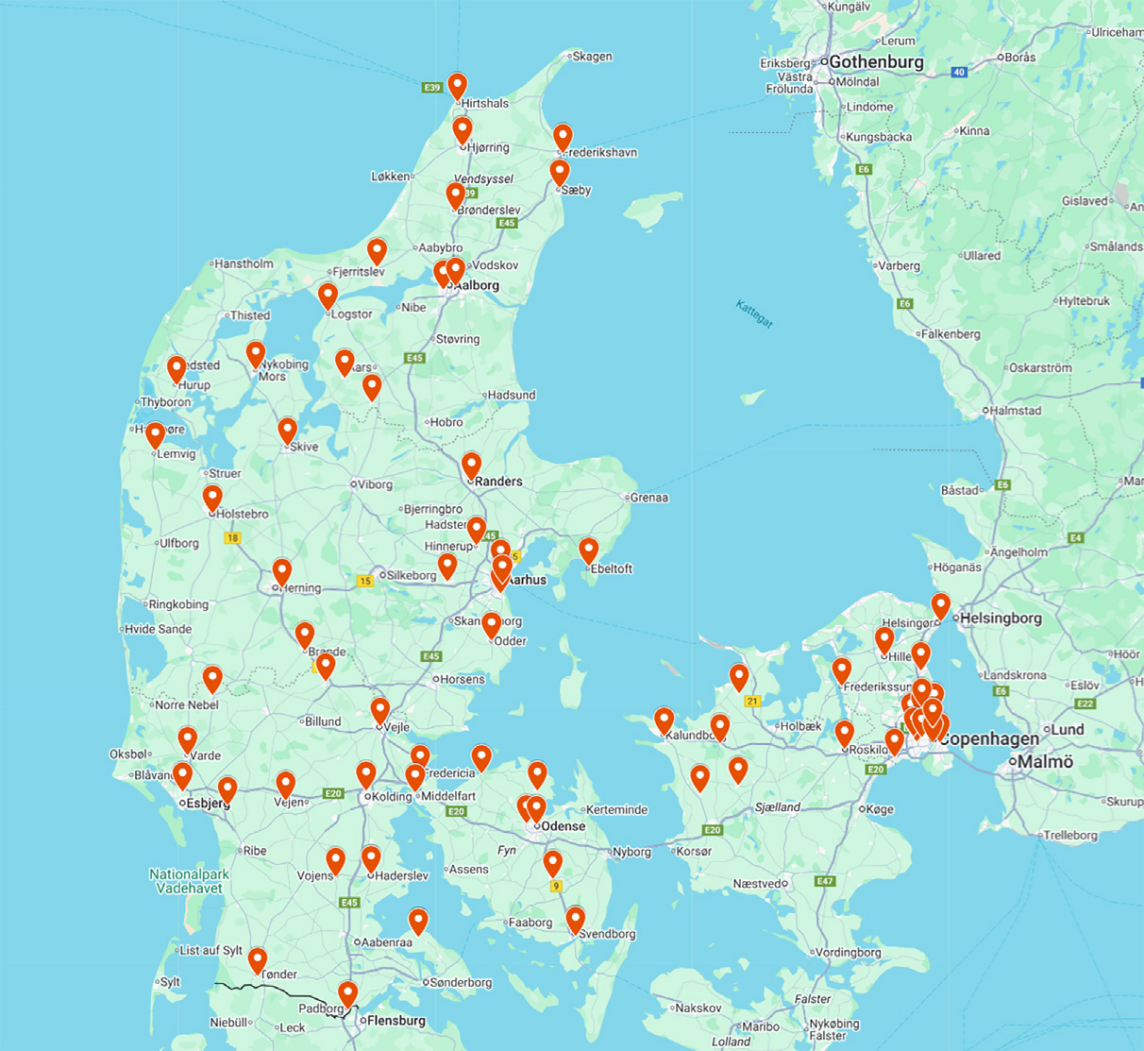
Locations providing packaging for this dataset. This figure illustrates the locations from which the samples of returned cosmetic and personal hygiene plastic packaging were sent to the retailer’s headquarters and subsequently delivered to our lab for analysis. The distribution of locations illustrates the geographic representativeness of the dataset for the continuous landmass of Denmark (i.e., the peninsula of Jutland and the Danish archipelago, excluding Bornholm, Greenland, and the Faroe Islands).

At present, consumers receive no incentive for returning goods to the store (Matas, 2025), nor were the consumers in any way informed of the effort to sample and analyse the collected materials. To return packaging, the consumer places the packaging in a dedicated bin at their local stores. Usually, these bags would be sent directly to the recycler with other waste arisings, but for our sampling, these bags were instead shipped back to the retailer's headquarters. The retailer then delivered the still-packed samples, including the geographic location of the collection point, via the attached shipping labels. The lab team had no direct contact with the sampling locations or local retailers; however, local interviews were conducted at retailers close to the location of the lab to ensure a holistic understanding of the collection system and common practices of the retail staff after the customer returns the packaging. As Fig. 1 illustrates, this dataset exhibits geographic representativeness of the continuous landmass of Denmark (i.e., the peninsula of Jutland and the Danish archipelago excluding Bornholm, Greenland, and the Faroe Islands), as samples were provided from urban, suburban, and rural areas, ensuring coverage of the major population centres in the peninsula of Jutland and the Danish archipelago.

### 4.1 Waste sampling procedure

The returned packaging waste was analysed between February and May 2025 at the Waste Laboratory in the Department of Resource and Environmental Engineering at the Technical University of Denmark. All samples were characterized via a four-tiered approach (level I-IV). The four tiers are: Tier I - Brand ownership; Tier II - Packaging design; Tier III - Product content; Tier IV - Container colour. Table 1 provides a detailed overview of each of these tiers. This multi-tiered approach enabled a comprehensive understanding of the packaging characteristics relevant for potential future studies on evaluating design patterns, consumer use, and potential implications for recyclability. Great care was taken in the naming convention to avoid misleading labels. The authors aimed to remain as faithful as possible to the nomenclature provided by the retailer. However, no standardized classification system for Tier II was identified, neither at the company level nor in the literature Table 4.

**Table 4:** Overview of four-tiered approach used for characterizing cosmetic and personal hygiene packaging waste. Each packaging sample was categorized according to four tiers: Tier I – Brand ownership (distinguishing private-label and external brands); Tier II – Packaging design (including both container and closure types); Tier III – Sample content type (classified based on expected emptying efficiency); and Tier IV – Container colour.

Tier I - Brand ownership	Tier II - Packaging design	Tier III - Product content	Tier IV - Container colour
**Primary private-label brand**	**Primary container types**	**Low emptying efficiency**	Beige
Matas Striber	Bottle	Aqueous solution	Black
**Other private-label brands**	Jar	Body scrub	Blue
e.g. Matas Natur	Tube	Cleaning detergent	Bronze
**External brands**	Others	Conditioner	Brown
e.g. Pharma Nord	**Closure mechanism**	Cosmetic	Clear
	Airless pump	Cream	Clear beige
	Disc-top cap	Deodorant	Clear blue
	Dropper caps	Facial cleanser	Clear green
	Fine mist sprayer	Gel	Clear pink
	Flip-top lid	Hair treatment	Dark blue
	Flip-top lid	Lip balm	Gold
	Foaming pump	Mousse	Green
	Lotion pump	Oil	Grey
	Nozzel	Paste	Light blue
	Roll-on lid	Perfume	Light brown
	Screw cap	Serum	Light green
	Serum pump	Soap	Light grey
	Sifter lid	Sunscreen	Mint green
	Snap-on cap	Travel size	Orange
	Snap-on lid	**High emptying efficiency**	Pink
	Tamper-evident lid	Bandage	Purple
		Cotton swab	Red
		Supplement	Semi-clear
		Powder	Silver

The brand ownership tier separates out the brand of the retailer from the distributed brands. As the in-house brand is the target of the scheme, this distinction enables tailored feedback for the retailer to improve the circularity of its own products. This also provides insight into the collection efficiency of the take-back scheme.

The second tier separates samples based on the structural packaging design, capturing both the main container and closure type. The final design assigned to a packaging product corresponds to the combination of the associated container and closure type. These design features are central to determining the recyclability of packaging, particularly in relation to mono- vs. multi-material configurations, ease of cleaning, and mechanical separability of components.

The third tier classifies products based on their emptying efficiency, as either low or high, referring to the content originally present in the packaging. This was distinguished based on product labels and visual inspection. Packaging requirements vary substantially depending on many factors, such as whether the content is liquid or non-liquid. This affects the polymer classification, presence of pumps or sprays, and likelihood of residual content - all of which influence recycling outcomes. Therefore, this tier provides insight into how different product formulations influence packaging, affect washability, and result in residual content.

At the final tier, each sample was characterized by the visible colour of the packaging, with special attention to black and dark-coloured plastics, which are often undetectable by optical sorting systems such as near-infrared (NIR) scanners and thus more likely to disrupt recycling.

### 4.2 Characterization procedure

**Fig. 2:**
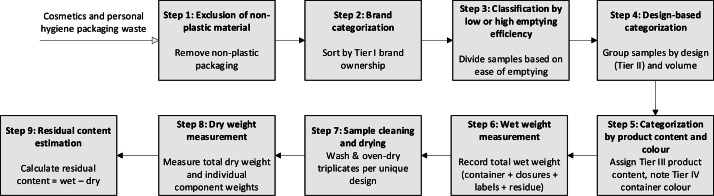
Nine-step characterization approach adopted in this study to characterize the collected cosmetics and personal-hygiene plastic packaging waste.

This study employed a systematic waste characterization approach, consistent with common practice for solid waste analysis [7,8]. The goal is to reproduce a replicable and detailed dataset. Every sample went through a nine-step characterization scheme, summarized in Fig. 2.

### Step 1: Exclusion of non-plastic material

The process involved transferring samples onto a clean, impermeable tarpaulin to prevent material loss and cross-contamination, followed by manual coarse sorting of the 270 kg bulk sample to isolate plastic packaging from missorted items, i.e. metal, glass, paper, the retailer’s gift cards and undefined materials. The missorted items accounted for approximately 22.5 kg, while the remaining packaging waste, where the primary container was made of plastic, was further analyzed in the subsequent steps of the waste characterization process.

### Step 2: Brand categorization

The retained plastic packaging was divided into three main categories according to tier I:

— Primary private-label brand: packaging from the retailer’s primary private-label product line which is the main consumer-facing brand i.e., Matas Striber.
— Other private-label brands: plastic packaging from the retailer’s additional in-house brands such as Matas Natur and Matas Material.
— External brands: plastic packaging from third-party cosmetic brands (i.e., all other manufacturers). The name of each manufacturer was reported.

### Step 3: Classification by low or high emptying efficiency

Samples were then divided into two groups, low and high emptying efficiency, based on the expected ease of removing the residual content from the packaging prior to cleaning.

### Step 4: Design-based categorization

The samples were further grouped by their design for the primary container and closure type according to tier II. Within each packaging format, samples were additionally sorted by declared volume.

### Step 5: Categorization by product content and colour

Items were grouped based on their product content as indicated on the label (e.g., shampoo, conditioner, deodorant), see tier III. During this fine sorting step, the colour of each container (excluding label or closure colour) was also noted (see tier IV), as it can be relevant for recycling considerations, though colour was not used to physically sort the items.

Each unique packaging design configuration obtained by a specific combination of packaging container and closure, product content, container colour and declared volume, was assigned a Group identification number (Group ID), and each individual sample in that group was given a “Sub-group ID”. Grouping was based on an identical set of key attributes: brand (e.g., retailer vs. external manufacturer, and specific product line), product content (e.g., soap, cream), packaging design (container and closure type), declared volume, and container colour.

### Step 6: Wet weight measurement

The total wet weight of each sample (container, residual content, closures, and labels) was weighed using an electronic scale.

### Step 7: Sample cleaning and drying

From each unique packaging design, three containers (triplicates) were selected when possible, opened, emptied and washed thoroughly with warm water, and dried in a lab oven at 105 °C for a minimum of 16 h in accordance with DS/EN 15,934. High emptying efficiency samples were generally easy to empty completely with no residues left inside; therefore, these containers did not undergo this step of the waste characterization analysis.

### Step 8: Dry weight measurement

The total dry weight of each cleaned sample was measured. Moreover, single components of each packaging unit such as caps and labels were also weighted individually.

### Step 9: Residual content estimation

The residual content for each sample was estimated as the difference between the wet and dry weight and then normalized by the container’s volume (g/ml). In cases of >3 samples for each unique packaging design combination, the mean of the samples from step 8 was used to calculate the residue content for the other samples.

All this information was recorded systematically in an Excel sheet. The measured data are presented as mean and standard deviation, unless otherwise indicated. The standardized classification system, developed to catalogue the sorted samples consistently was derived from a pilot project conducted prior to the main waste characterization campaign. This allowed the research team to test the procedure, identify practical challenges, and refine the methodology before scaling up.

## Limitations

The sample consisted of packaging collected from 72 retail locations across Denmark (out of a total of 263 shops) between February through March 2025. This may mean the study does not capture the real recycling rate, nor the variations in seasonal products, which may otherwise have been present in the sample gathered at a different time of year. Therefore, this dataset should be viewed as representative of the weights and residual content of the sampled materials but not used as a representative sample of the collection efficiencies or capacity of retail takeback systems for cosmetic or personal-hygiene packaging. Thus, there may also be seasonal fluctuations in product use, particularly sunscreen, and user behaviour (e.g., discretionary discarding at year-end) that could alter residue levels in future sampling efforts.

Furthermore, as the take-back system is operated by a single retailer, the findings primarily reflect the retailer’s own product lines. This limits the generalizability of the results beyond that brand context and prevents a comprehensive understanding of the broader cosmetic packaging waste stream, particularly for products sold through other retail channels or disposed of via municipal systems.

Finally, the behaviours and trends that lead to residual content are influenced not only by packaging design but also by consumer practices and habits associated with cosmetic product use. Since the data reflect only those consumers who actively participate in the Matas take-back scheme, the sample likely captures individuals with above-average environmental awareness or engagement. This introduces a behavioural bias that may affect the observed levels of residual content, and should be considered when interpreting the dataset’s applicability to the general population or to systems with different consumer profiles.

## Ethics Statement

The authors have read and followed the ethical requirements for publication in Data in Brief. We confirm that the current work does not involve human subjects, animal experiments, or any data from social media platforms.

## CRediT Author Statement

**Gloria Moscatelli:** participated in the methodology, investigation, validation, formal analysis, data curation, visualization, writing - original draftwriting - review & editing, and editing of the dataset and manuscript. She also provided lab supervision throughout the characterization study. **Heather Margaret Logan:** participated in the conceptualization, methodology, project administration, data validation, data curation, visualization, writing - review & editing, and supervision of the dataset and the final manuscript. **Lærke Djurhuus Ipsen:** participated in the methodology, investigation, and validation of the dataset. **Jonathan Holger Harlev Astrup:** participated in the methodology, investigation, and validation of the dataset. **Jakob Seidel Bisgaard:** participated in the methodology, investigation, and validation of the dataset. **Ricardo Gabbay de Souza:** participated in the provision of resources, writing - review & editing and supervision of the lab work involved in the waste characterization. He also participated in the reviewing and editing of the final manuscript. **Marie Kampmann Eriksen:** participated in the conceptualization, methodological development, and supervision of the project. **Anders Damgaard:** participated in the conceptualization, supervision, and funding acquisition of the project. He also contributed to data curation as well as writing - review & editing the final manuscript.

## Data Availability

Technical University of Denmark Data RepositoryCosmetics and Personal Hygiene Primary Plastic Packaging Dataset: Characterization of Post-Consumer Waste Returned via Take-back System in Denmark (Original data).

## Appendix Supplementary materials

Image, application 1
