# Supplementary material for: Cosmetics and personal hygiene primary plastic packaging dataset: Characterization of post-consumer waste returned via take-back system in Denmark
Source: Data Brief. 2025 Nov 23;64:112300. doi: 10.1016/j.dib.2025.112300 (PMC12881732; doi:10.1016/j.dib.2025.112300)
Supplement: Supplementary file 1 [file mmc1.zip › SupplementarymaterialS1Packagingdesign.pdf]

Supplementary material S1 – Packaging design: standardized coding scheme for container and closure types, including reference photos of packaging designs

| **Primary container type - CODE** | **Primary container type** | **Specific closure mechanism type - CODE** | **Closure mechanism type** | **Photo** |
|---|---|---|---|---|
| B | Bottle | SC | screw_cap | 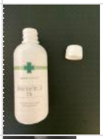 |
| B | Bottle | SCO | screw_cap_over | 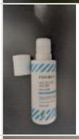 |
| B | Bottle | SCT | screw_cap_traditional | 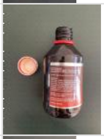 |
| B | Bottle | SCS | screw_cap_safety | 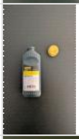 |
| B | Bottle | FTL | fliptop_lid | 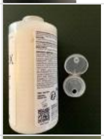 |
| B | Bottle | SCH | screw_cap_hole | 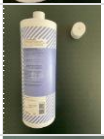 |
| B | Bottle | DTC | disctop_cap | 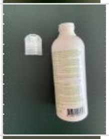 |
| B | Bottle | N | nozzel | 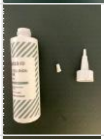 |
| B | Bottle | DC | dropper_caps | 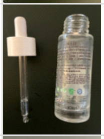 |
| B | Bottle | APO | airless_pump_w_overcap | 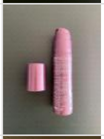 |
| B | Bottle | APT | airless_pump_twist | 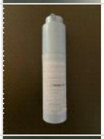 |
| B | Bottle | FPO | foaming_pump_w_overcap | 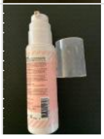 |

| | | | | |
|---|---|---|---|---|
| B | Bottle | LP | lotion_pump | 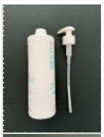 |
| B | Bottle | LPT | lotion_pump_traditional | 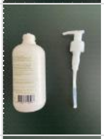 |
| B | Bottle | SP | serum_pump | 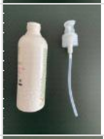 |
| B | Bottle | APSO | airless_pump_small_w_overcap | 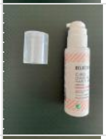 |
| B | Bottle | FMSO | fine_mist_sprayer_w_overcap | 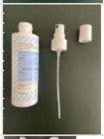 |
| B | Bottle | FMS | fine_mist_sprayer | 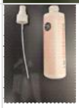 |
| J | Jar | SCC | screw_cap_cross | 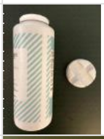 |
| J | Jar | SCSP | screw_cap_safety_pills | 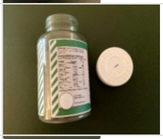 |
| J | Jar | SCA | screw_cap_arrow | 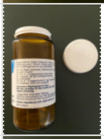 |
| J | Jar | FTLR | fliptop_lid_round | 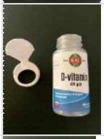 |
| J | Jar | TEL | tamperevident_lid | 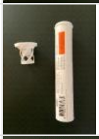 |
| J | Jar | SCF | screw _cap_flat | 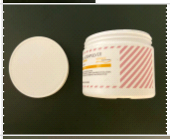 |
| J | Jar | TEL | tamperevident_lid | 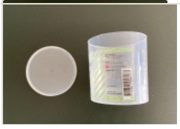 |

| | | | | |
|---|---|---|---|---|
| J | Jar | SC | screw_cap | 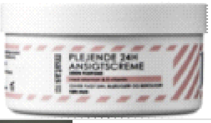  |
| J | Jar | SCS | screw_cap_safety | 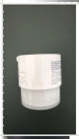 |
| J | Jar | SCT | screw_cap_traditional | 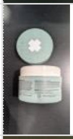 |
| J | Jar | SCF | screw_cap_flat | 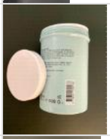 |
| T | Tube | FTL | fliptop_lid | 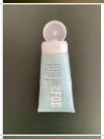 |
| T | Tube | SC | screw_cap | 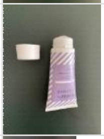 |
| T | Tube | PO | pump_w_overcap | 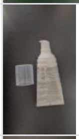 |
| T | Tube | SCT | screw cap top | 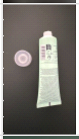 |
| S | Others_Sachet | NA | na | 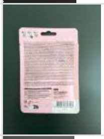 |
| B | Others_Bottle | SOC | snapon_cap | 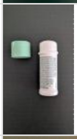 |
| B | Others_Bottle | ROO | rollon_lid_w_overcap | 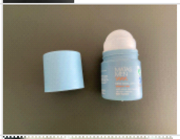 |
| B | Others_Bottle | SL | sifter_lid | 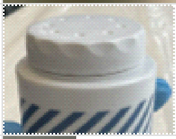 |
| C | Others_Compact | SC | screw_cap | 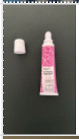 |
| PEN | Others_Pencil_Barrel | SOL | snapon_lid | 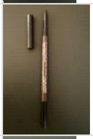 |
| C | Others_Compact | SCWA | screw_cap_w_wand_applicator | 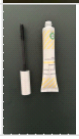 |
